# Causal Associations Between Gut Microbes and Heart Failure Across Multiple Etiologies: A Mendelian Randomization Study

**DOI:** 10.31083/RCM46534

**Published:** 2026-01-22

**Authors:** Xinming Xu, Safraz Anwar, Yunpeng Shang, Xiaogang Guo, Xiao Cui

**Affiliations:** ^1^Department of Cardiology, The First Affiliated Hospital, Zhejiang University School of Medicine, 311121 Hangzhou, Zhejiang, China; ^2^Graduate School, Zhejiang University School of Medicine, 310029 Hangzhou, Zhejiang, China

**Keywords:** gut microbes, heart failure, *Peptostreptococcaceae*, hypertensive heart diseases

## Abstract

**Background:**

Gut microbiota are associated with heart failure (HF); however, the causal relationship between gut microbial communities and HF of varying etiologies remains incompletely established.

**Methods:**

This study leveraged two-sample Mendelian randomization (MR) to investigate whether genetically determined gut microbiota features causally influence HF and its related subtypes. Instrumental variables (IVs) for gut microbiota were derived from a large-scale, genome-wide association study (GWAS) of microbial traits conducted by the MiBioGen consortium, which included 18,340 individuals. Summary statistics for HF and its subtypes were extracted from the FinnGen Release 7, encompassing 19,350 all-cause HF cases and 288,996 controls. The Wald ratio and inverse-variance weighted analyses were applied to calculate the causal estimates.

**Results:**

A total of 19 single-nucleotide polymorphisms (SNPs) corresponding to 18 gut microbial taxa were selected as IVs. A significant inverse causal association was identified between the family *Peptostreptococcaceae* and the risk of hypertensive heart disease (odds ratio (OR): 0.355, 95% confidence interval (CI): 0.193–0.656; *p* < 0.001; q = 0.018). Several additional taxa showed suggestive causal associations with HF or its precursor conditions, although these did not survive multiple-testing correction.

**Conclusions:**

Genetically predicted enrichment of *Peptostreptococcaceae* is causally associated with a lower risk of hypertensive heart disease. These MR findings warrant a mechanistic dissection of *Peptostreptococcaceae*-mediated pathways as a potential therapeutic lever for the prevention and treatment of hypertension-mediated HF.

## 1. Introduction 

Heart failure (HF) is the terminal stage of diverse cardiovascular disorders and is defined by structural or functional cardiac abnormalities that generate elevated intracardiac pressures and/or insufficient cardiac output [1]. HF affects approximately 1–2% of adults globally [1, 2, 3], and arises from a wide spectrum of cardiovascular conditions, including coronary artery disease, hypertension, valvular heart disease, and cardiomyopathies. The predominant etiological factors vary geographically and temporally, collectively imposing a major global disease burden [4].

In addition to conventional cardiac drivers, the role of the gut in the initiation and progression of HF has gained increasing recognition [5]. Patients with HF exhibit consistent, disease-specific shifts in the gut microbiota composition, which exceed the variability observed in healthy aging. Proposed mechanistic links include splanchnic hypoperfusion, barrier disruption, and bacterial translocation, yet contemporary research has increasingly focused on the implications of gut microbiota dysbiosis [6, 7, 8]. In particular, microbial metabolites that can modulate myocardial energetics, systemic inflammation, and vascular tone have attracted widespread interest for their potentially protective or detrimental roles in HF and other cardiovascular conditions [6, 9, 10, 11]. However, observational studies yield conflicting results, with the directionality and magnitude of the association differing across cohorts [12, 13]. These discrepancies likely stem from uncontrolled environmental and clinical confounders, reverse causation (microbial changes as a consequence rather than a cause of HF), and compositional heterogeneity of the microbiome [14]. Therefore, robust evidence that disentangles causality from correlation is required. Although randomized controlled trials (RCTs) are considered the gold standard for inferring causality, implementing these studies in this context is challenging due to the complex interactions between the gut microbiome and host biology, as well as their resource-intensive, time-consuming, and ethically complex nature.

Mendelian randomization (MR) offers a complementary, genetically anchored approach for assessing causality [15]. The rationale of MR is to use instrumental variables (IVs) as proxies to explore causal inferences between exposures and the diseases of interest, mostly single-nucleotide polymorphisms (SNPs), which are identified from genome-wide association studies (GWASs). Moreover, based on valid IVs, MR estimates are less biased by confounding factors and not susceptible to reverse causation [16]. Hence, by leveraging the summary statistics of the most recent large-scale gut microbiome trait loci (mbTL) and HF GWAS datasets, we conducted a two-sample MR study to evaluate the causal relationship between gut microbiota and HF, including its subtype-related conditions.

## 2. Materials and Methods

### 2.1 Study Design

**Fig. 1.**
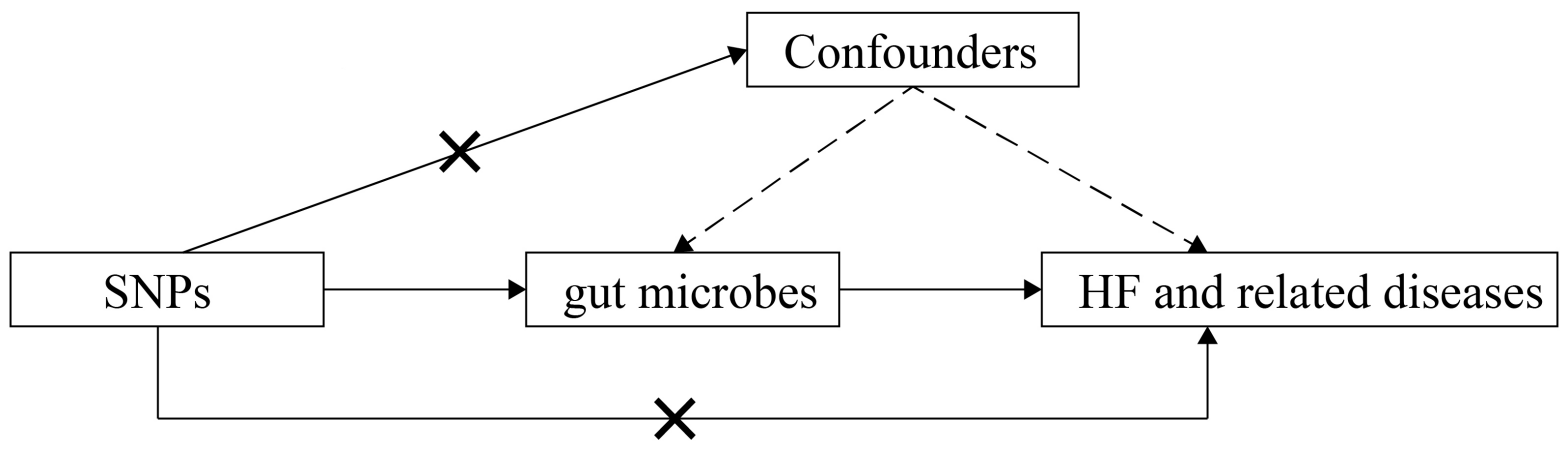
**Schematic representation of this MR study**. SNPs, single-nucleotide polymorphisms; HF, heart failure; MR, Mendelian randomization.

Utilizing summary data from published studies and corresponding resources, as detailed below, we performed a two-sample MR study to evaluate the causal relationship between gut microbiota and HF of various etiologies (Fig. 1).

### 2.2 Selection of Instrumental Variables

IVs for gut microbes were identified from host genetic loci associated with the microbiome at a genome-wide significance threshold (*p* < 5 × 10^-8^), based on large-scale association analyses of genome-wide genotypes and 16S fecal microbiome data from 18,340 individuals conducted by the MiBioGen consortium (https://mibiogen.gcc.rug.nl/) [17, 18]. All selected IVs satisfied the three core assumptions necessary for a valid MR analysis: (1) IVs that are strongly associated with the modifiable exposure of interest (gut microbes); (2) IVs that are independent of any confounders influencing both the exposure and the outcome; (3) IVs that affect the outcome solely through the exposure, with no direct or alternative pathways [19]. Linkage disequilibrium clumping (r^2^ < 0.01; 10,000 kb window) was performed to ensure allelic independence. Palindromic SNPs with ambiguous strand alignment (A/T or C/G) were excluded.

### 2.3 Data Sources of Outcomes

The GWAS summary statistics for HF and the associated diseases were obtained from FinnGen Release 7, one of the pioneering personalized medicine projects designed to elucidate genotype–phenotype correlations by aggregating and analyzing genomic and health registry data from Finnish biobank participants (https://www.finngen.fi/en). Detailed GWAS processing methods are described in the documentation (https://finngen.gitbook.io/documentation/v/r7/methods/phewas). Covariates used in the model included sex, age, 10 principal components, and genotyping batch. The case and control numbers for each outcome were as follows: all-cause HF, 19,350 cases and 288,996 controls; hypertensive heart disease, 6348 cases and 223,663 controls; coronary heart disease with HF, 9463 cases and 275,526 controls; valvular heart disease excluding rheumatic fever, 62,218 cases and 218,984 controls; cardiomyopathy, 4606 cases and 218,984 controls; infective endocarditis, 700 cases and 218,984 controls; pulmonary heart disease, 7450 cases and 301,704 controls.

Meanwhile, the case and control numbers among the subtypes of cardiomyopathies were as follows: non-ischemic cardiomyopathy, 7047 cases and 253,401 controls; primary cardiomyopathy, 3238 cases and 218,984 controls; hypertrophic cardiomyopathy, 808 cases and 308,346 controls; hypertrophic cardiomyopathy with HF, 326 cases and 308,346 controls; alcoholic cardiomyopathy, 99 cases and 309,055 controls.

Finally, the case and control numbers among the subtypes of valvular heart diseases were as follows: non-rheumatic valve disease, 15,799 cases and 218,984 controls; rheumatic valve disease, 575 cases and 308,346 controls; calcific aortic valvular stenosis, 6870 cases and 302,284 controls.

Outcome definitions were based on nationwide registries and harmonized according to the International Classification of Diseases (ICD) revisions 8, 9, and 10, as well as ICD-O-3 for oncology, NOMESCO procedure codes, drug reimbursement codes from the Finnish Social Insurance Institution (KELA), and Anatomical Therapeutic Chemical (ATC) codes (https://www.finngen.fi/en/researchers/clinical-endpoints). An additional independent GWAS dataset for hypertensive heart disease was obtained from the U.S. Department of Veterans Affairs Million Veteran Program and used in the validation analysis: 60,962 cases and 358,617 controls of European ancestry (https://www.ebi.ac.uk/gwas/, GWAS Catalog, GCST90475924).

### 2.4 Statistical Analysis

All analyses were two-tailed; statistical significance was set at α = 0.05. The *p*-values were adjusted for multiple comparisons using the Benjamini-Hochberg procedure and reported as q-values. The strength of the instrumental variables was assessed using F-statistics derived from the first-stage regression of exposure on IVs, computed via the approximation method [20]. Wald ratio testing was applied for estimates involving a single IV. Otherwise, the inverse-variance weighted (IVW) method was conducted based on the fixed-effects model. All analyses were performed with R version 4.2.1 (http://www.r-project.org), using the “TwoSampleMR” and “MRPRESSO” packages.

## 3. Results

### 3.1 Basic Characteristics of IVs for Gut Microbes

A total of 19 independent SNPs served as IVs for 18 gut microbial taxa (**Supplementary Table 1**). Among these independent SNPs, two (rs7322849 and rs182549) were associated with the genus *Bifidobacterium*, while each of the remaining microbes was associated with a unique SNP. The F-statistic values ranged from 29.812 to 88.430, exceeding the conventional threshold of 10 and indicating that the instruments possess adequate strength to yield reliable causal inferences.

### 3.2 Genetic Proxied GM Abundance on HF With Various Causes

**Fig. 2.**
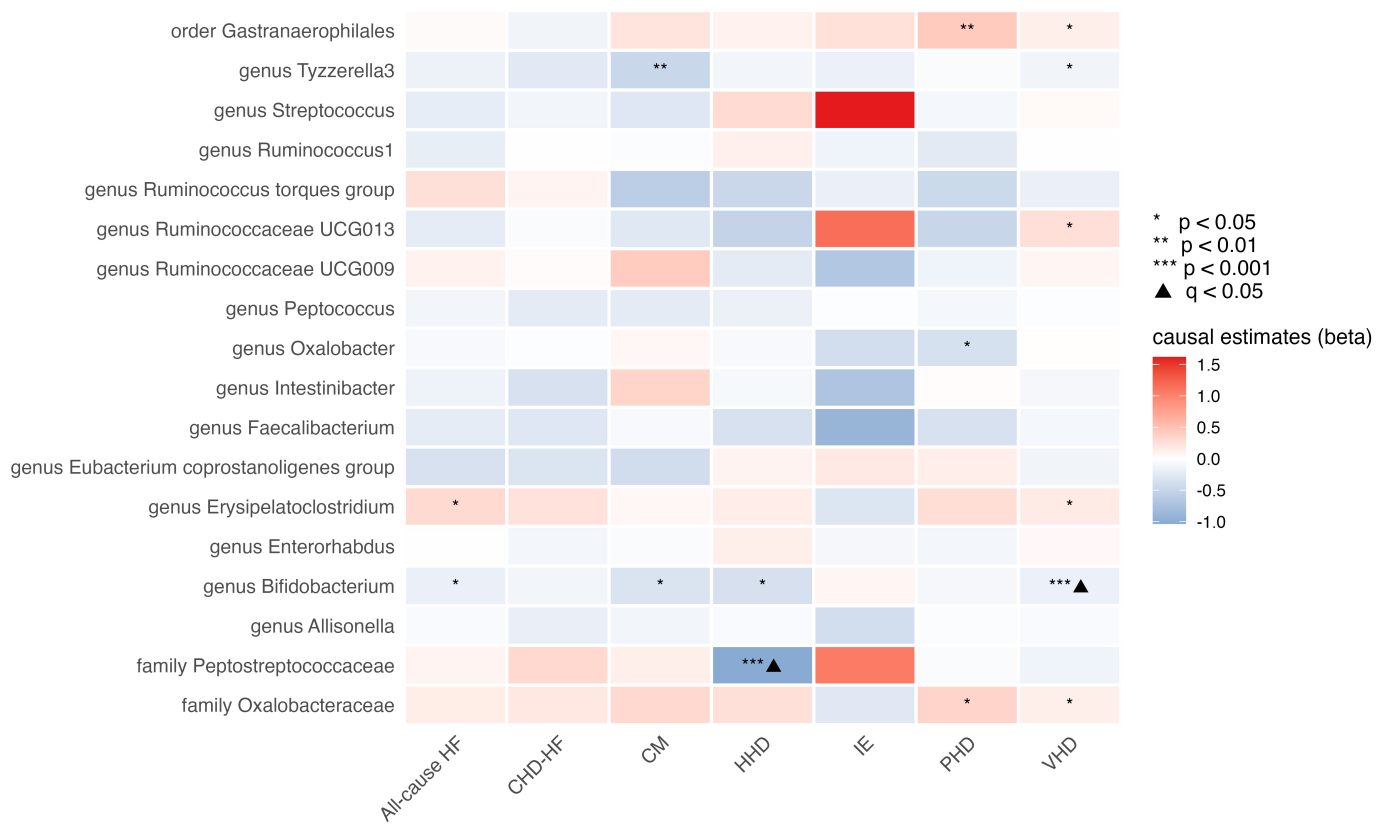
**Heatmap of the causal estimates of genetically proxied gut microbe abundance on the risks of HF with various causes**. The black triangle signified a statistically significant finding after the correction for multiple testing (q-value < 0.05). CHD–HF, coronary heart disease with HF; CM, cardiomyopathy; HHD, hypertensive heart disease; IE, infective endocarditis; PHD, pulmonary heart disease; VHD, valvular heart disease excluding rheumatic fever.

Fig. 2 summarises the MR estimates for 18 microbial taxa across HF phenotypes. After conducting the correction for multiple testing, the genetically determined enrichment of the family *Peptostreptococcaceae* was associated with a 64.5% reduction in the risk of hypertensive heart disease (odds ratio (OR): 0.355, 95% confidence interval (CI): 0.193–0.656; *p* < 0.001; q = 0.018; Table 1), supporting a causal protective effect. To validate this finding, we further utilized an additional independent GWAS dataset for hypertensive heart disease from the Million Veteran Program. This independent analysis revealed a negative association between the family *Peptostreptococcaceae* and hypertensive heart disease, although this association did not reach statistical significance (OR: 0.897, 95% CI: 0.724–1.113; *p* = 0.325). Next, we conducted a reverse MR analysis to elucidate the directionality of the causal association between the family *Peptostreptococcaceae* and hypertensive heart disease. Six independent SNPs functioned as IVs for hypertensive heart disease, with F-statistics ranging from 29.994 to 45.310 (**Supplementary Table 2**). The reverse MR analysis indicated that genetically determined hypertensive heart disease was not significantly associated with *Peptostreptococcaceae* abundance (OR: 1.042, 95% CI: 0.957–1.135; *p* = 0.340), suggesting no significant reverse causal effects.

**Table 1.** **Significant or suggestive causal associations between gut microbes and HF and HF-related diseases based on two-sample MR analyses**.

Exposure	Outcome	Method	Number of SNPs	Beta	OR (95% CI)	*p*-value	q-value
Genus *Erysipelatoclostridium*	All-cause HF	Wald ratio	1	0.301	1.352 (1.039–1.759)	0.025	0.309
Genus *Bifidobacterium*	All-cause HF	IVW	2	–0.181	0.834 (0.707–0.985)	0.033	0.309
Family *Peptostreptococcaceae*	HHD	Wald ratio	1	–1.034	0.355 (0.193–0.656)	0.001	0.018
Genus *Bifidobacterium*	HHD	IVW	2	–0.366	0.693 (0.524–0.917)	0.010	0.098
Genus *Ruminococcaceae UCG013*	VHD	Wald ratio	1	0.261	1.299 (1.026–1.643)	0.030	0.101
Genus *Tyzzerella3*	VHD	Wald ratio	1	–0.134	0.875 (0.780–0.981)	0.022	0.101
Order *Gastranaerophilales*	VHD	Wald ratio	1	0.133	1.143 (1.016–1.285)	0.027	0.101
Genus *Erysipelatoclostridium*	VHD	Wald ratio	1	0.167	1.182 (1.007–1.386)	0.040	0.109
Genus *Bifidobacterium*	VHD	IVW	2	–0.169	0.844 (0.763–0.934)	0.001	0.009
Family *Oxalobacteraceae*	VHD	Wald ratio	1	0.134	1.143 (1.012–1.292)	0.032	0.101
Genus *Tyzzerella3*	CM	Wald ratio	1	–0.478	0.620 (0.432–0.890)	0.010	0.182
Genus *Bifidobacterium*	CM	IVW	2	–0.337	0.714 (0.521–0.979)	0.036	0.230
Family *Oxalobacteraceae*	PHD	Wald ratio	1	0.353	1.424 (1.055–1.921)	0.021	0.132
Order *Gastranaerophilales*	PHD	Wald ratio	1	0.429	1.536 (1.145–2.060)	0.004	0.079
Genus *Oxalobacter*	PHD	Wald ratio	1	–0.361	0.697 (0.526–0.923)	0.012	0.113
Genus *Bifidobacterium*	NICM	IVW	2	–0.287	0.750 (0.579–0.973)	0.030	0.287
Family *Oxalobacteraceae*	PCM	Wald ratio	1	0.500	1.648 (1.046–2.597)	0.031	0.209
Genus *Tyzzerella3*	PCM	Wald ratio	1	–0.464	0.629 (0.410–0.963)	0.033	0.209
Genus *Ruminococcaceae UCG009*	PCM	Wald ratio	1	0.724	2.063 (1.224–3.476)	0.007	0.124
Genus *Intestinibacter*	HCM	Wald ratio	1	1.821	6.178 (1.532–24.904)	0.010	0.199
Genus *Streptococcus*	HCM	Wald ratio	1	–1.838	0.159 (0.031–0.828)	0.029	0.274
Family *Oxalobacteraceae*	HCM–HF	Wald ratio	1	1.406	4.079 (1.000–16.631)	0.050	0.475
Genus *Enterorhabdus*	HCM–HF	Wald ratio	1	–1.757	0.173 (0.035–0.851)	0.031	0.475
Genus *Allisonella*	NRVD	Wald ratio	1	–0.217	0.805 (0.683–0.948)	0.009	0.177
Genus *Tyzzerella3*	RVD	Wald ratio	1	–1.318	0.268 (0.098–0.730)	0.010	0.191
Genus *peptococcus*	CAVS	Wald ratio	1	–0.406	0.666 (0.480–0.924)	0.015	0.144
Family *Oxalobacteraceae*	CAVS	Wald ratio	1	–0.411	0.663 (0.483–0.910)	0.011	0.144
Genus *Allisonella*	CAVS	Wald ratio	1	–0.247	0.781 (0.618–0.987)	0.038	0.242

IVW, inverse-variance weighted (fixed effects); OR, odds ratio; CI, confidence interval; NICM, non-ischemic cardiomyopathy; PCM, primary cardiomyopathy; HCM, hypertrophic cardiomyopathy; HCM–HF, hypertrophic cardiomyopathy with HF; NRVD, non-rheumatic valve disease; RVD, rheumatic valve disease; CAVS, calcific aortic valvular stenosis.

Several additional taxa also showed associations with HF and related conditions, although these did not reach statistical significance after multiple testing correction, indicating suggestive causal links. For instance, the genus *Erysipelatoclostridium* was positively correlated with all-cause HF (OR: 1.352, 95% CI: 1.039–1.759; *p* = 0.025; q = 0.309; Table 1), whereas the *Bifidobacterium* exhibited a negative association (OR: 0.834, 95% CI: 0.707–0.985; *p* = 0.033; q = 0.309; Table 1). Additionally, the genus *Tyzzerella3* was inversely associated with cardiomyopathy (OR: 0.620, 95% CI: 0.432–0.890; *p* = 0.010; q = 0.182; Table 1). Suggestive causal associations were also observed between pulmonary heart disease and the order Gastranaerophilales (OR: 1.536, 95% CI: 1.145–2.060; *p* = 0.004; q = 0.079), the family *Oxalobacteraceae* (OR: 1.424, 95% CI: 1.055–1.921; *p* = 0.021; q = 0.132), and the genus *Oxalobacter* (OR: 0.697, 95% CI: 0.526–0.923, *p* = 0.012, q = 0.113; all in Table 1). Furthermore, the genus *Allisonella* demonstrated a suggestive inverse association with non-rheumatic valvular heart disease (OR: 0.805, 95% CI: 0.683–0.948; *p* = 0.009; q = 0.177; Table 1). The complete MR results are provided in **Supplementary Table 3**.

### 3.3 Genetic Proxied GM Abundance on Cardiomyopathy Subtypes

**Fig. 3.**
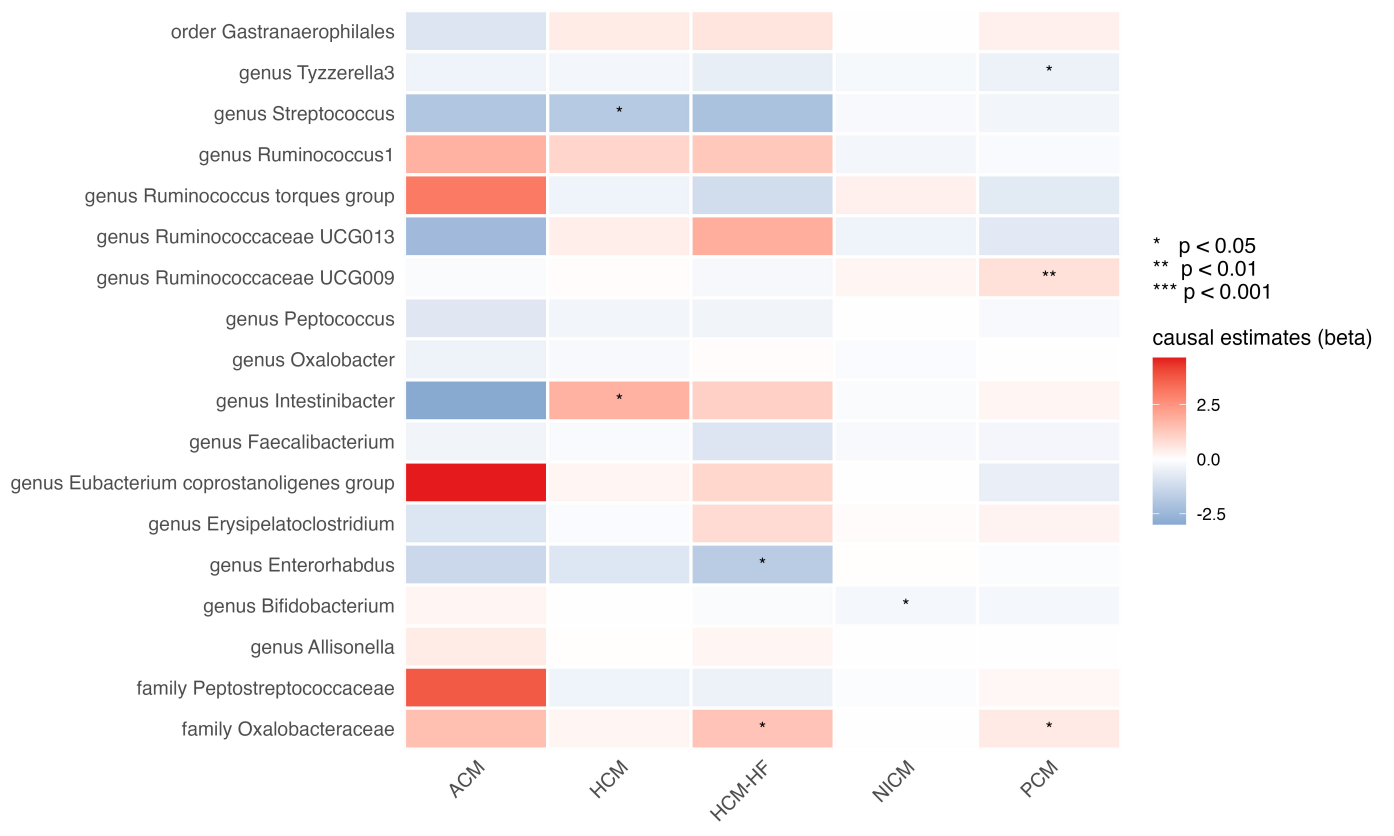
**Heatmap of the causal estimates of genetically proxied gut microbe abundance on the risks of cardiomyopathy subtypes**. ACM, alcoholic cardiomyopathy; NICM, non-ischemic cardiomyopathy.

Cardiomyopathies comprise a heterogeneous group of myocardial disorders with divergent pathophysiology. Therefore, we interrogated whether individual microbial taxa exert etiology-specific effects across predefined subtypes. After employing a false discovery rate (FDR) correction, no associations reached statistical significance (Fig. 3). However, several suggestive causal associations were observed. The genus *Bifidobacterium* exhibited an inverse association with non-ischemic cardiomyopathy (OR: 0.750, 95% CI: 0.579–0.973; *p* = 0.030; q = 0.287; Table 1). Meanwhile, suggestive associations with primary cardiomyopathy were detected for the family *Oxalobacteraceae* (OR: 1.648, 95% CI: 1.046–2.597; *p* = 0.031; q = 0.209), the genus *Tyzzerella3* (OR: 0.629, 95% CI: 0.410–0.963; *p* = 0.033; q = 0.209), and the genus *Ruminococcaceae* UCG009 (OR: 2.063, 95% CI: 1.224–3.476; *p* = 0.007; q = 0.124; all in Table 1). Notably, the Genus *Intestinibacter* showed suggestive causal effects on hypertrophic cardiomyopathy (OR: 6.178, 95% CI: 1.532–24.904; *p* = 0.010; q = 0.199; Table 1), whereas the genus *Streptococcus* demonstrated a protective trend (OR: 0.159, 95% CI: 0.031–0.828; *p* = 0.029; q = 0.274; Table 1). However, the related gut microbes are quite different for patients with hypertrophic cardiomyopathy complicated by HF than those with hypertrophic cardiomyopathy alone. The high OR for the family *Oxalobacteraceae*, which shows a suggestive causal relationship with primary cardiomyopathy, also revealed a correlation with hypertrophic cardiomyopathy with HF (OR: 4.079, 95% CI: 1.000–16.631; *p* < 0.050; q = 0.475; Table 1). Meanwhile, the genus *Enterorhabdus* tended to decrease the risk of hypertrophic cardiomyopathy with HF (OR: 0.173, 95% CI: 0.035–0.851; *p* = 0.031; q = 0.475; Table 1). The complete MR results for the cardiomyopathy subtypes are provided in **Supplementary Table 4**.

### 3.4 Genetic Proxied GM Abundance on Valvular Heart Disease Subtypes

**Fig. 4.**
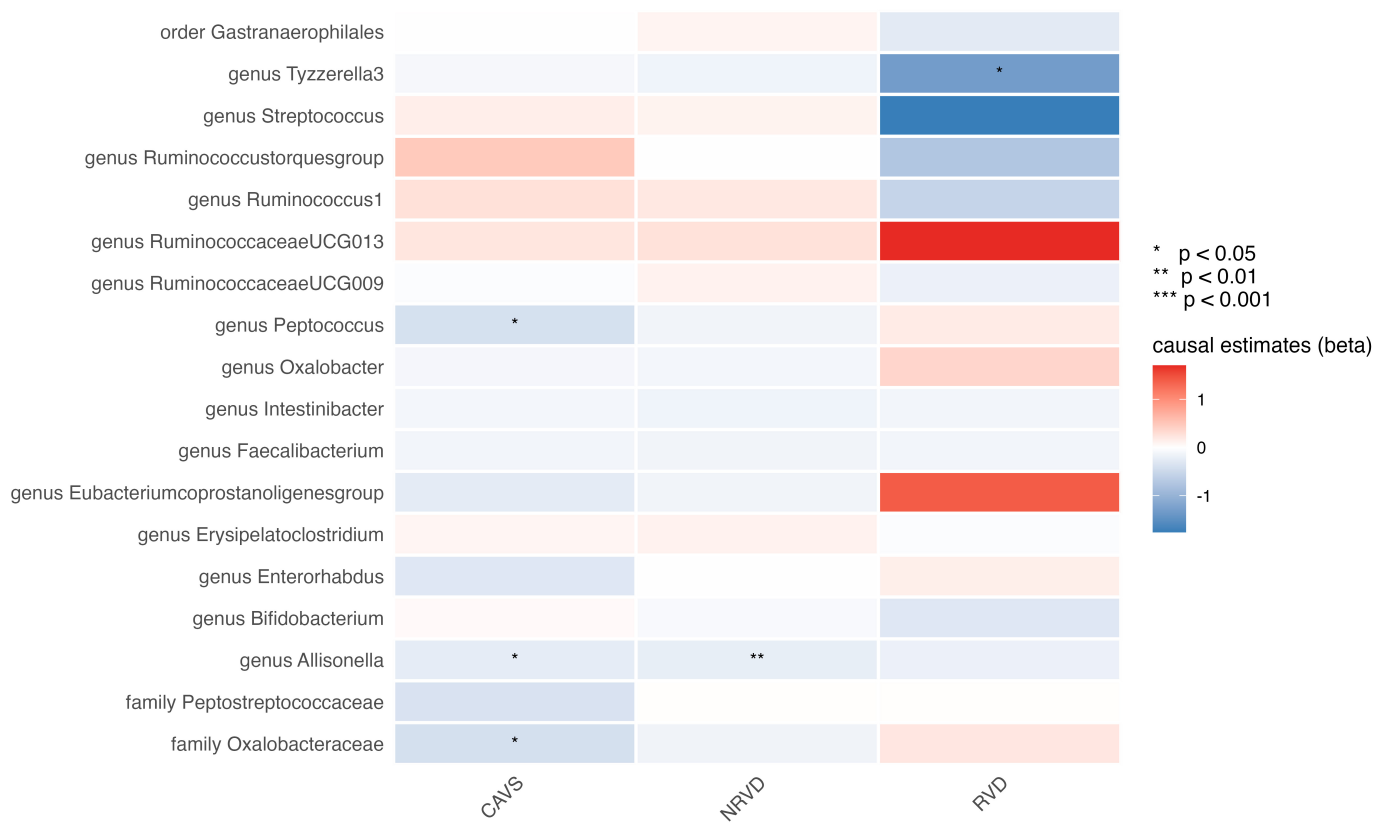
**Heatmap of the causal estimates of genetically proxied gut microbe abundance on the risks of valvular heart disease subtypes**.

Given the importance of valvular heart disease (VHD) as an etiology of HF, we examined causal relationships with three major VHD subtypes: non-rheumatic valve diseases, rheumatic valve diseases, and calcific aortic valvular stenosis. As shown in Fig. 4, no associations were noted following multiple testing correction (Fig. 4); however, several taxa displayed consistent directional signals. The genus *Allisonella* showed inverse associations with both non-rheumatic valve diseases (OR: 0.805, 95% CI: 0.683–0.948; *p* = 0.009; q = 0.177) and calcific aortic valvular stenosis (OR: 0.781, 95% CI: 0.618–0.987; *p* = 0.038; q = 0.242; Table 1). Similarly, the genus *Peptococcus* (OR: 0.666, 95% CI: 0.480–0.924; *p* = 0.015; q = 0.144) and the family *Oxalobacteraceae* (OR: 0.663, 95% CI: 0.483–0.910; *p* = 0.011; q = 0.144) were inversely associated with calcific aortic valvular stenosis (Table 1). The genus *Tyzzerella3*, previously implicated in cardiomyopathy, was also inversely related to rheumatic valve diseases (OR: 0.268, 95% CI: 0.098–0.730; *p* = 0.010; q = 0.191; Table 1). The complete MR results for the VHD subtypes are listed in **Supplementary Table 5**.

## 4. Discussion

This study investigated causal relationships between gut microbiota composition and HF of diverse etiologies using a two-sample MR approach with independent IVs. After adjusting for multiple comparisons, we obtained robust evidence that genetically predicted enrichment of the family *Peptostreptococcaceae* is associated with a lower risk of hypertensive heart disease. Additionally, several suggestive causal associations were observed between specific microbial taxa and HF-related conditions.

Our MR analysis suggests a protective association between the family *Peptostreptococcaceae* and hypertensive heart disease, although the underlying causal mechanisms are currently unknown. One speculative pathway, based on prior literature, could involve microbial metabolites. Previous studies demonstrated that the family *Peptostreptococcaceae* is positively associated with trimethylamine-N-oxide (TMAO) production [21]. Meanwhile, TMAO has multiple biological functions, particularly as a well-known detrimental molecule in cardiovascular diseases [22, 23, 24, 25]. However, emerging evidence is currently challenging this perception, prompting a significant paradigm shift in understanding the multifaceted roles of TMAO. Indeed, initial observational studies have linked elevated circulating TMAO levels to an increased risk of atherosclerosis, a finding supported by preclinical models demonstrating adverse effects of supraphysiological TMAO doses in atherosclerosis-prone mice and on thrombus formation [23, 26, 27, 28]. Conversely, subsequent investigations in both human cohorts and murine models have failed to corroborate these initial associations and even suggested that TMAO exerts a protective effect in atherosclerosis [29, 30]. Additionally, some analyses indicated that the previously reported negative correlation might be confounded by renal function, as the association disappeared after adjusting for this variable through appropriate statistical correction, suggesting that elevated TMAO levels might primarily reflect impaired renal excretion rather than directly contributing to disease pathogenesis [31, 32, 33]. The causative role of TMAO in cardiovascular pathology has become increasingly recognized as context-dependent, influenced by a multitude of factors, including concentration, the physiological state of the host, and interactions with other microbial and host pathways [34, 35, 36]. Moreover, a growing body of recent research has revealed protective functions for TMAO. Additionally, prior evidence has suggested that TMAO could exert protective effects, including stabilizing proteins and cells during osmotic stress under specific pathophysiological conditions [37, 38, 39]. The beneficial effects of TMAO have been documented in diseases such as hypertension and non-alcoholic steatohepatitis, as well as in promoting glucose tolerance and blood–brain barrier integrity [40, 41, 42]. Meanwhile, chronic low-dose TMAO administration in hypertensive rat models reduced cardiac fibrosis, ventricular pressure, and biomarkers of cardiac dysfunction, such as NH2-terminal pro-B-type natriuretic peptide and vasopressin [40]. This finding aligns with the hypothesis that TMAO elevation might represent a compensatory host response rather than a primary maladaptation against pressure overload and high-salt-induced osmotic stress [43]. However, it is still too premature to attribute the possible mechanistic associations of *Peptostreptococcaceae* against hypertensive heart disease to this speculation. Thus, future work should move beyond correlation analyses and prioritize functional experiments designed to directly test whether and how *Peptostreptococcaceae* influences cardiac remodeling in hypertension.

Building upon our current findings, further elucidating the causal effect of *Peptostreptococcaceae* on hypertensive heart disease and its specific underlying mechanisms holds significant translational potential for developing novel therapeutic and preventive strategies. From a practical perspective, this would necessitate the development of targeted modulators, such as next-generation probiotics or prebiotics designed to selectively promote the growth and certain metabolic activity of beneficial taxa within the family *Peptostreptococcaceae*. The implementation of such interventions would likely follow a stratified medicine approach, where patients are first screened for a low abundance of these bacteria, thereby identifying those most likely to benefit from treatment. Perspectively, integrating these findings into patient care would require a structured translational pathway. The adoption of this strategy would depend on the subsequent additive benefit within the existing treatment paradigm and its cost-effectiveness, potentially adding a new, microbiome-targeted dimension to the management of hypertensive heart disease.

In addition to *Peptostreptococcaceae*, we detected consistent but non-significant trends implicating the genus *Bifidobacterium* in the protection mechanisms against all-cause HF, hypertensive heart disease, cardiomyopathy, and its subset of non-ischemic cardiomyopathies. This is consistent with the established role of *Bifidobacterium* as a health-associated commensal and its therapeutic investigation as a probiotic for cardiovascular diseases [44, 45, 46, 47]. Conversely, the genus *Tyzzerella3* showed suggestive inverse associations with rheumatic valve disease and primary cardiomyopathy. While prior studies have found *Tyzzerella* to be enriched in individuals with high lifetime cardiovascular disease risks and transverse aortic constriction models, our MR analysis suggests a potentially protective relationship and warrants further study [48, 49]. These discrepancies underscore the limitations of observational designs in disentangling cause from consequence and highlight the value of genetic instrumentation in microbiome research. The family *Oxalobacteraceae* exhibited particularly intriguing, phenotype-specific effects: suggestive positive effects on pulmonary heart disease, primary cardiomyopathy, and hypertrophic cardiomyopathy with HF, but inverse effects on calcific aortic stenosis. Such divergent estimates imply that gut microbes may exert divergent or even opposing influences across distinct cardiac pathophysiologies, underscoring the need to explore microbial interactions and disease-specific mechanisms.

Several limitations should be acknowledged. First, associations failing to reach statistical significance for many taxa do not definitively exclude causality, because statistical power is constrained by the finite number of cases and the relatively small variance explained by lead SNPs. Second, restriction to European ancestry populations limits generalizability to other ethnic groups. Meanwhile, although these datasets are derived from independent consortia, the potential for an unknown degree of sample overlap cannot be entirely excluded. Hence, future validations in independent populations, such as those of Asian ancestry, are needed. Third, the complexity of host genetics–microbiome interactions and the limited impact of genetic variants on microbial taxa variability necessitate cautious interpretation of causality estimates. Fourth, the limited number of IVs for each gut microbial taxon might reduce the statistical power and affect the robustness of the causal estimates. Future studies based on microbiome GWAS on a larger scale are needed to provide more powerful instruments and validate our findings. Fifth, although the core GWAS analyses by FinnGen have already been adjusted for covariates, including age, sex, the first 10 principal components, and genotyping batch, known confounding factors such as kidney function, medication use, and diet may still introduce bias. Finally, our analysis focused on taxonomic relative abundance; therefore, future work incorporating metagenomic or metatranscriptomic functional profiles might provide deeper insights into the biochemical pathways underpinning the observed associations. Functional investigations in larger studies and diverse methodologies, such as gnotobiotic models and clinical trials, are essential before therapeutic translation.

## 5. Conclusions

In summary, this MR study provided genetically based evidence supporting a causal, protective role of *Peptostreptococcaceae* against hypertensive heart disease, highlighting the potential values of this family and its metabolites as predictive biomarkers and therapeutic targets for pressure-overload-related cardiac remodeling. The suggestive but non-significant trends observed for multiple additional taxa furnish a comprehensive catalog of hypotheses that can be prioritized in experimental and clinical microbiome studies. Ultimately, integrating genetic instrumentation with mechanistic and interventional research is required to determine whether precision manipulation of the gut microbiota can prevent or treat the diverse syndromes that culminate in HF.

## Availability of Data and Materials

Publicly available datasets were analyzed in this study. This data can be found here: MiBioGen consortium (www.mibiogen.org) and FinnGen release 7 (https://finngen.gitbook.io/documentation/v/r7/).

## Supplementary Material

Supplementary material associated with this article can be found, in the online version, at https://doi.org/10.31083/RCM46534.
