# Supplementary material for: Causal Associations Between Gut Microbes and Heart Failure Across Multiple Etiologies: A Mendelian Randomization Study
Source: Rev Cardiovasc Med. 2026 Jan 22;27(1):46534. doi: 10.31083/RCM46534 (PMC12873699; doi:10.31083/RCM46534)
Supplement: Supplementary file 1 [file 2153-8174-27-1-46534-s1.zip › Supplementary Material.docx]

**Supplementary Materials**

**Supplementary Table 1.** Genetic instrumental variables and effect values of gut microbes used for two-sample MR analyses

| **exposure** | **SNP** | **chromosome** | **EA** | **OA** | **beta** | **se** | **p value** | **appF** |
| --- | --- | --- | --- | --- | --- | --- | --- | --- |
| order *Gastranaerophilales* | rs9864379 | 3 | T | C | -0.161 | 0.029 | 4.66E-08 | 30.065 |
| family *Oxalobacteraceae* | rs4428215 | 3 | G | A | 0.126 | 0.023 | 4.88E-08 | 29.812 |
| family *Peptostreptococcaceae* | rs61841503 | 10 | G | A | 0.092 | 0.016 | 9.8E-09 | 32.495 |
| genus *Eubacterium coprostanoligenes group* | rs17159861 | 7 | C | T | 0.096 | 0.017 | 1.04E-08 | 32.654 |
| genus *Ruminococcus torques group* | rs35866622 | 19 | T | C | -0.061 | 0.011 | 2.21E-08 | 31.285 |
| genus *Allisonella* | rs602075 | 9 | A | G | 0.169 | 0.030 | 3.57E-08 | 32.374 |
| genus *Bifidobacterium* | rs7322849 | 13 | T | C | 0.112 | 0.020 | 1.08E-08 | 31.035 |
| genus *Bifidobacterium* | rs182549 | 2 | C | T | 0.120 | 0.013 | 1.28E-20 | 88.430 |
| genus *Enterorhabdus* | rs11098863 | 4 | T | A | -0.097 | 0.016 | 3.06E-09 | 35.262 |
| genus *Erysipelatoclostridium* | rs7221249 | 17 | A | G | 0.084 | 0.014 | 4.31E-09 | 34.619 |
| genus *Faecalibacterium* | rs12320842 | 12 | C | G | 0.095 | 0.016 | 7.57E-09 | 33.453 |
| genus *Intestinibacter* | rs10805326 | 4 | G | A | 0.078 | 0.014 | 3.55E-08 | 30.803 |
| genus *Oxalobacter* | rs736744 | 9 | C | T | 0.118 | 0.021 | 2.57E-08 | 31.135 |
| genus *Peptococcus* | rs75754569 | 3 | C | G | 0.181 | 0.032 | 1.1E-08 | 32.262 |
| genus *Ruminococcaceae UCG009* | rs8009993 | 14 | G | C | -0.136 | 0.024 | 4.42E-08 | 30.821 |
| genus *Ruminococcaceae UCG013* | rs12781711 | 10 | C | T | -0.066 | 0.012 | 2.55E-08 | 31.194 |
| genus *Ruminococcus1* | rs10769159 | 11 | G | C | -0.064 | 0.011 | 5.29E-09 | 33.844 |
| genus *Streptococcus* | rs11110281 | 12 | T | C | -0.138 | 0.023 | 2.58E-09 | 36.572 |
| genus *Tyzzerella3* | rs67476743 | 19 | T | G | 0.132 | 0.022 | 3.74E-09 | 35.417 |

SNPs, single-nucleotide polymorphisms; MR, Mendelian randomization; EA, effect allele; OA, other alleles.

**Supplementary Table 2.** Genetic instrumental variables and effect values of hypertensive heart disease used for the reverse MR analysis

| **SNP** | **chromosome** | **EA** | **OA** | **beta** | **se** | **p value** | **appF** |
| --- | --- | --- | --- | --- | --- | --- | --- |
| rs17035646 | 1 | A | G | 0.129 | 0.019 | 1.68E-11 | 45.310 |
| rs1759693 | 1 | T | C | 0.126 | 0.022 | 1.65E-08 | 31.872 |
| rs1275985 | 2 | T | C | -0.123 | 0.019 | 8.10E-11 | 42.234 |
| rs17477177 | 7 | C | T | 0.112 | 0.020 | 4.33E-08 | 29.994 |
| rs3918226 | 7 | T | C | 0.212 | 0.036 | 4.71E-09 | 34.304 |
| rs661348 | 11 | C | T | 0.125 | 0.019 | 3.49E-11 | 43.882 |

SNPs, single-nucleotide polymorphisms; MR, Mendelian randomization; EA, effect allele; OA, other alleles.

**Supplementary Table 3.** Causal estimates of genetic proxied gut microbe abundance on the risks of heart failure with various causes based on two-sample MR analyses

| **exposure** | **outcome** | **method** | **nSNP** | **beta** | **OR (95%CI)** | **p value** | **q value** |
| --- | --- | --- | --- | --- | --- | --- | --- |
| family *Peptostreptococcaceae* | All-cause HF | Wald ratio | 1 | 0.091 | 1.095 (0.766-1.567) | 0.619 | 0.691 |
| family *Oxalobacteraceae* | All-cause HF | Wald ratio | 1 | 0.148 | 1.160 (0.949-1.418) | 0.148 | 0.447 |
| genus *Peptococcus* | All-cause HF | Wald ratio | 1 | -0.114 | 0.892 (0.724-1.099) | 0.282 | 0.447 |
| genus *Intestinibacter* | All-cause HF | Wald ratio | 1 | -0.154 | 0.858 (0.627-1.172) | 0.335 | 0.487 |
| genus *Enterorhabdus* | All-cause HF | Wald ratio | 1 | -0.016 | 0.985 (0.784-1.236) | 0.893 | 0.893 |
| genus *Erysipelatoclostridium* | All-cause HF | Wald ratio | 1 | 0.301 | 1.352 (1.039-1.759) | 0.025 | 0.309 |
| genus *Tyzzerella3* | All-cause HF | Wald ratio | 1 | -0.155 | 0.856 (0.709-1.034) | 0.107 | 0.447 |
| order *Gastranaerophilales* | All-cause HF | Wald ratio | 1 | 0.038 | 1.039 (0.856-1.262) | 0.699 | 0.738 |
| genus *Bifidobacterium* | All-cause HF | IVW | 2 | -0.181 | 0.834 (0.707-0.985) | 0.033 | 0.309 |
| genus *Oxalobacter* | All-cause HF | Wald ratio | 1 | -0.074 | 0.929 (0.769-1.122) | 0.446 | 0.564 |
| genus *Allisonella* | All-cause HF | Wald ratio | 1 | -0.053 | 0.948 (0.818-1.100) | 0.483 | 0.574 |
| genus *Ruminococcaceae UCG009* | All-cause HF | Wald ratio | 1 | 0.106 | 1.112 (0.886-1.395) | 0.359 | 0.487 |
| genus *Faecalibacterium* | All-cause HF | Wald ratio | 1 | -0.227 | 0.797 (0.566-1.121) | 0.192 | 0.447 |
| genus *Streptococcus* | All-cause HF | Wald ratio | 1 | -0.213 | 0.808 (0.558-1.171) | 0.259 | 0.447 |
| genus *Ruminococcaceae UCG013* | All-cause HF | Wald ratio | 1 | -0.222 | 0.801 (0.543-1.180) | 0.261 | 0.447 |
| genus *Eubacterium coprostanoligenes group* | All-cause HF | Wald ratio | 1 | -0.356 | 0.701 (0.483-1.016) | 0.061 | 0.384 |
| genus *Ruminococcus1* | All-cause HF | Wald ratio | 1 | -0.207 | 0.813 (0.572-1.156) | 0.249 | 0.447 |
| genus *Ruminococcus torques group* | All-cause HF | Wald ratio | 1 | 0.254 | 1.289 (0.883-1.881) | 0.188 | 0.447 |
| family *Peptostreptococcaceae* | HHD | Wald ratio | 1 | -1.034 | 0.355 (0.193-0.656) | 0.001 | 0.018 |
| family *Oxalobacteraceae* | HHD | Wald ratio | 1 | 0.257 | 1.293 (0.921-1.813) | 0.137 | 0.555 |
| genus *Peptococcus* | HHD | Wald ratio | 1 | -0.173 | 0.841 (0.591-1.197) | 0.337 | 0.703 |
| genus *Intestinibacter* | HHD | Wald ratio | 1 | -0.085 | 0.919 (0.543-1.555) | 0.752 | 0.758 |
| genus *Enterorhabdus* | HHD | Wald ratio | 1 | 0.128 | 1.136 (0.774-1.667) | 0.515 | 0.703 |
| genus *Erysipelatoclostridium* | HHD | Wald ratio | 1 | 0.146 | 1.157 (0.743-1.802) | 0.518 | 0.703 |
| genus *Tyzzerella3* | HHD | Wald ratio | 1 | -0.112 | 0.894 (0.651-1.230) | 0.492 | 0.703 |
| order *Gastranaerophilales* | HHD | Wald ratio | 1 | 0.110 | 1.116 (0.805-1.548) | 0.510 | 0.703 |
| genus *Bifidobacterium* | HHD | IVW | 2 | -0.366 | 0.693 (0.524-0.917) | 0.010 | 0.098 |
| genus *Oxalobacter* | HHD | Wald ratio | 1 | -0.066 | 0.936 (0.681-1.287) | 0.685 | 0.758 |
| genus *Allisonella* | HHD | Wald ratio | 1 | -0.060 | 0.942 (0.734-1.209) | 0.639 | 0.758 |
| genus *Ruminococcaceae UCG009* | HHD | Wald ratio | 1 | -0.238 | 0.788 (0.537-1.157) | 0.224 | 0.608 |
| genus *Faecalibacterium* | HHD | Wald ratio | 1 | -0.357 | 0.700 (0.393-1.245) | 0.224 | 0.608 |
| genus *Streptococcus* | HHD | Wald ratio | 1 | 0.300 | 1.349 (0.725-2.513) | 0.345 | 0.703 |
| genus *Ruminococcaceae UCG013* | HHD | Wald ratio | 1 | -0.523 | 0.593 (0.309-1.137) | 0.116 | 0.555 |
| genus *Eubacterium coprostanoligenes group* | HHD | Wald ratio | 1 | 0.098 | 1.103 (0.590-2.063) | 0.758 | 0.758 |
| genus *Ruminococcus1* | HHD | Wald ratio | 1 | 0.127 | 1.136 (0.629-2.049) | 0.673 | 0.758 |
| genus *Ruminococcus torques group* | HHD | Wald ratio | 1 | -0.473 | 0.623 (0.330-1.179) | 0.146 | 0.555 |
| family *Peptostreptococcaceae* | CHD-HF | Wald ratio | 1 | 0.314 | 1.369 (0.814-2.305) | 0.237 | 0.571 |
| family *Oxalobacteraceae* | CHD-HF | Wald ratio | 1 | 0.200 | 1.221 (0.912-1.634) | 0.180 | 0.571 |
| genus *Peptococcus* | CHD-HF | Wald ratio | 1 | -0.236 | 0.789 (0.583-1.068) | 0.126 | 0.571 |
| genus *Intestinibacter* | CHD-HF | Wald ratio | 1 | -0.358 | 0.699 (0.444-1.101) | 0.122 | 0.571 |
| genus *Enterorhabdus* | CHD-HF | Wald ratio | 1 | -0.110 | 0.896 (0.643-1.247) | 0.514 | 0.751 |
| genus *Erysipelatoclostridium* | CHD-HF | Wald ratio | 1 | 0.236 | 1.266 (0.863-1.857) | 0.228 | 0.571 |
| genus *Tyzzerella3* | CHD-HF | Wald ratio | 1 | -0.262 | 0.770 (0.585-1.013) | 0.062 | 0.571 |
| order *Gastranaerophilales* | CHD-HF | Wald ratio | 1 | -0.128 | 0.880 (0.664-1.168) | 0.376 | 0.596 |
| genus *Bifidobacterium* | CHD-HF | IVW | 2 | -0.112 | 0.894 (0.702-1.137) | 0.361 | 0.596 |
| genus *Oxalobacter* | CHD-HF | Wald ratio | 1 | -0.025 | 0.975 (0.741-1.283) | 0.856 | 0.926 |
| genus *Allisonella* | CHD-HF | Wald ratio | 1 | -0.198 | 0.820 (0.661-1.017) | 0.071 | 0.571 |
| genus *Ruminococcaceae UCG009* | CHD-HF | Wald ratio | 1 | 0.032 | 1.033 (0.743-1.436) | 0.847 | 0.926 |
| genus *Faecalibacterium* | CHD-HF | Wald ratio | 1 | -0.280 | 0.756 (0.460-1.243) | 0.271 | 0.571 |
| genus *Streptococcus* | CHD-HF | Wald ratio | 1 | -0.124 | 0.884 (0.514-1.520) | 0.655 | 0.889 |
| genus *Ruminococcaceae UCG013* | CHD-HF | Wald ratio | 1 | -0.044 | 0.957 (0.544-1.681) | 0.878 | 0.926 |
| genus *Eubacterium coprostanoligenes group* | CHD-HF | Wald ratio | 1 | -0.324 | 0.723 (0.421-1.244) | 0.241 | 0.571 |
| genus *Ruminococcus1* | CHD-HF | Wald ratio | 1 | -0.011 | 0.989 (0.594-1.649) | 0.967 | 0.967 |
| genus *Ruminococcus torques group* | CHD-HF | Wald ratio | 1 | 0.091 | 1.095 (0.633-1.896) | 0.746 | 0.926 |
| genus *Intestinibacter* | VHD | Wald ratio | 1 | -0.091 | 0.913 (0.755-1.103) | 0.346 | 0.487 |
| genus *Ruminococcaceae UCG013* | VHD | Wald ratio | 1 | 0.261 | 1.299 (1.026-1.643) | 0.030 | 0.101 |
| genus *Ruminococcus torques group* | VHD | Wald ratio | 1 | -0.178 | 0.837 (0.665-1.053) | 0.129 | 0.306 |
| genus *Faecalibacterium* | VHD | Wald ratio | 1 | -0.097 | 0.908 (0.738-1.116) | 0.359 | 0.487 |
| genus *Allisonella* | VHD | Wald ratio | 1 | -0.054 | 0.947 (0.866-1.037) | 0.239 | 0.423 |
| genus *Streptococcus* | VHD | Wald ratio | 1 | 0.044 | 1.045 (0.835-1.308) | 0.701 | 0.784 |
| genus *Tyzzerella3* | VHD | Wald ratio | 1 | -0.134 | 0.875 (0.780-0.981) | 0.022 | 0.101 |
| genus *Ruminococcaceae UCG009* | VHD | Wald ratio | 1 | 0.078 | 1.081 (0.942-1.241) | 0.267 | 0.423 |
| genus *Eubacterium coprostanoligenes group* | VHD | Wald ratio | 1 | -0.131 | 0.877 (0.700-1.099) | 0.254 | 0.423 |
| order *Gastranaerophilales* | VHD | Wald ratio | 1 | 0.133 | 1.143 (1.016-1.285) | 0.027 | 0.101 |
| genus *Erysipelatoclostridium* | VHD | Wald ratio | 1 | 0.167 | 1.182 (1.007-1.386) | 0.040 | 0.109 |
| genus *Oxalobacter* | VHD | Wald ratio | 1 | 0.017 | 1.017 (0.907-1.140) | 0.773 | 0.816 |
| genus *Peptococcus* | VHD | Wald ratio | 1 | -0.025 | 0.975 (0.859-1.107) | 0.695 | 0.784 |
| genus *Bifidobacterium* | VHD | IVW | 2 | -0.169 | 0.844 (0.763-0.934) | 0.001 | 0.009 |
| genus *Ruminococcus1* | VHD | Wald ratio | 1 | -0.010 | 0.990 (0.800-1.226) | 0.928 | 0.928 |
| genus *Enterorhabdus* | VHD | Wald ratio | 1 | 0.054 | 1.056 (0.919-1.212) | 0.442 | 0.560 |
| family *Oxalobacteraceae* | VHD | Wald ratio | 1 | 0.134 | 1.143 (1.012-1.292) | 0.032 | 0.101 |
| family *Peptostreptococcaceae* | VHD | Wald ratio | 1 | -0.140 | 0.870 (0.700-1.080) | 0.207 | 0.423 |
| family *Peptostreptococcaceae* | CM | Wald ratio | 1 | 0.128 | 1.136 (0.575-2.245) | 0.713 | 0.889 |
| family *Oxalobacteraceae* | CM | Wald ratio | 1 | 0.317 | 1.373 (0.936-2.015) | 0.105 | 0.343 |
| genus *Peptococcus* | CM | Wald ratio | 1 | -0.233 | 0.792 (0.532-1.179) | 0.251 | 0.487 |
| genus *Intestinibacter* | CM | Wald ratio | 1 | 0.348 | 1.416 (0.780-2.571) | 0.253 | 0.487 |
| genus *Enterorhabdus* | CM | Wald ratio | 1 | -0.044 | 0.957 (0.620-1.478) | 0.843 | 0.889 |
| genus *Erysipelatoclostridium* | CM | Wald ratio | 1 | 0.065 | 1.067 (0.646-1.762) | 0.800 | 0.889 |
| genus *Tyzzerella3* | CM | Wald ratio | 1 | -0.478 | 0.620 (0.432-0.890) | 0.010 | 0.182 |
| order *Gastranaerophilales* | CM | Wald ratio | 1 | 0.224 | 1.251 (0.864-1.809) | 0.235 | 0.487 |
| genus *Bifidobacterium* | CM | IVW | 2 | -0.337 | 0.714 (0.521-0.979) | 0.036 | 0.230 |
| genus *Oxalobacter* | CM | Wald ratio | 1 | 0.068 | 1.070 (0.747-1.533) | 0.711 | 0.889 |
| genus *Allisonella* | CM | Wald ratio | 1 | -0.115 | 0.891 (0.672-1.182) | 0.425 | 0.673 |
| genus *Ruminococcaceae UCG009* | CM | Wald ratio | 1 | 0.423 | 1.526 (0.991-2.349) | 0.055 | 0.261 |
| genus *Faecalibacterium* | CM | Wald ratio | 1 | -0.075 | 0.928 (0.485-1.777) | 0.822 | 0.889 |
| genus *Streptococcus* | CM | Wald ratio | 1 | -0.280 | 0.756 (0.374-1.526) | 0.435 | 0.673 |
| genus *Ruminococcaceae UCG013* | CM | Wald ratio | 1 | -0.278 | 0.757 (0.361-1.585) | 0.461 | 0.673 |
| genus *Eubacterium coprostanoligenes group* | CM | Wald ratio | 1 | -0.409 | 0.664 (0.327-1.347) | 0.256 | 0.487 |
| genus *Ruminococcus1* | CM | Wald ratio | 1 | -0.032 | 0.969 (0.495-1.896) | 0.927 | 0.927 |
| genus *Ruminococcus torques group* | CM | Wald ratio | 1 | -0.593 | 0.553 (0.268-1.140) | 0.108 | 0.343 |
| genus *Enterorhabdus* | IE | Wald ratio | 1 | -0.092 | 0.912 (0.306-2.716) | 0.869 | 0.919 |
| family *Peptostreptococcaceae* | IE | Wald ratio | 1 | 1.067 | 2.907 (0.530-15.949) | 0.219 | 0.875 |
| genus *Streptococcus* | IE | Wald ratio | 1 | 1.616 | 5.033 (0.868-29.183) | 0.072 | 0.875 |
| genus *Ruminococcus torques group* | IE | Wald ratio | 1 | -0.184 | 0.832 (0.135-5.110) | 0.842 | 0.919 |
| order *Gastranaerophilales* | IE | Wald ratio | 1 | 0.251 | 1.285 (0.509-3.243) | 0.595 | 0.919 |
| genus *Peptococcus* | IE | Wald ratio | 1 | -0.026 | 0.974 (0.359-2.648) | 0.959 | 0.959 |
| genus *Bifidobacterium* | IE | IVW | 2 | 0.073 | 1.075 (0.487-2.376) | 0.857 | 0.919 |
| genus *Intestinibacter* | IE | Wald ratio | 1 | -0.714 | 0.490 (0.110-2.187) | 0.350 | 0.908 |
| family *Oxalobacteraceae* | IE | Wald ratio | 1 | -0.270 | 0.764 (0.291-2.000) | 0.583 | 0.919 |
| genus *Ruminococcaceae UCG009* | IE | Wald ratio | 1 | -0.677 | 0.508 (0.172-1.502) | 0.221 | 0.875 |
| genus *Faecalibacterium* | IE | Wald ratio | 1 | -0.908 | 0.403 (0.079-2.069) | 0.276 | 0.875 |
| genus *Ruminococcus1* | IE | Wald ratio | 1 | -0.141 | 0.869 (0.161-4.689) | 0.870 | 0.919 |
| genus *Allisonella* | IE | Wald ratio | 1 | -0.402 | 0.669 (0.330-1.359) | 0.266 | 0.875 |
| genus *Tyzzerella3* | IE | Wald ratio | 1 | -0.168 | 0.845 (0.341-2.094) | 0.716 | 0.919 |
| genus *Ruminococcaceae UCG013* | IE | Wald ratio | 1 | 1.159 | 3.187 (0.497-20.442) | 0.222 | 0.875 |
| genus *Oxalobacter* | IE | Wald ratio | 1 | -0.402 | 0.669 (0.271-1.649) | 0.382 | 0.908 |
| genus *Eubacterium coprostanoligenes group* | IE | Wald ratio | 1 | 0.178 | 1.194 (0.202-7.052) | 0.845 | 0.919 |
| genus *Erysipelatoclostridium* | IE | Wald ratio | 1 | -0.311 | 0.733 (0.208-2.577) | 0.628 | 0.919 |
| family *Peptostreptococcaceae* | PHD | Wald ratio | 1 | -0.044 | 0.957 (0.562-1.630) | 0.871 | 0.905 |
| family *Oxalobacteraceae* | PHD | Wald ratio | 1 | 0.353 | 1.424 (1.055-1.921) | 0.021 | 0.132 |
| genus *Peptococcus* | PHD | Wald ratio | 1 | -0.094 | 0.910 (0.667-1.241) | 0.552 | 0.807 |
| genus *Intestinibacter* | PHD | Wald ratio | 1 | 0.028 | 1.029 (0.646-1.639) | 0.905 | 0.905 |
| genus *Enterorhabdus* | PHD | Wald ratio | 1 | -0.111 | 0.895 (0.638-1.257) | 0.523 | 0.807 |
| genus *Erysipelatoclostridium* | PHD | Wald ratio | 1 | 0.273 | 1.314 (0.888-1.946) | 0.172 | 0.468 |
| genus *Tyzzerella3* | PHD | Wald ratio | 1 | -0.049 | 0.953 (0.719-1.262) | 0.735 | 0.834 |
| order *Gastranaerophilales* | PHD | Wald ratio | 1 | 0.429 | 1.536 (1.145-2.060) | 0.004 | 0.079 |
| genus *Bifidobacterium* | PHD | IVW | 2 | -0.089 | 0.915 (0.715-1.172) | 0.483 | 0.807 |
| genus *Oxalobacter* | PHD | Wald ratio | 1 | -0.361 | 0.697 (0.526-0.923) | 0.012 | 0.113 |
| genus *Allisonella* | PHD | Wald ratio | 1 | -0.036 | 0.964 (0.773-1.202) | 0.746 | 0.834 |
| genus *Ruminococcaceae UCG009* | PHD | Wald ratio | 1 | -0.143 | 0.867 (0.618-1.216) | 0.409 | 0.807 |
| genus *Faecalibacterium* | PHD | Wald ratio | 1 | -0.356 | 0.700 (0.422-1.164) | 0.169 | 0.468 |
| genus *Streptococcus* | PHD | Wald ratio | 1 | -0.097 | 0.907 (0.523-1.574) | 0.729 | 0.834 |
| genus *Ruminococcaceae UCG013* | PHD | Wald ratio | 1 | -0.489 | 0.613 (0.344-1.094) | 0.098 | 0.428 |
| genus *Eubacterium coprostanoligenes group* | PHD | Wald ratio | 1 | 0.142 | 1.153 (0.662-2.007) | 0.615 | 0.834 |
| genus *Ruminococcus1* | PHD | Wald ratio | 1 | -0.251 | 0.778 (0.461-1.314) | 0.348 | 0.807 |
| genus *Ruminococcus torques group* | PHD | Wald ratio | 1 | -0.457 | 0.633 (0.360-1.114) | 0.113 | 0.428 |

MR, Mendelian randomization; nSNP, number of single-nucleotide polymorphisms. IVW, inverse variance weighted (fixed effects); OR, odds ratio; CI, confidence interval; HF, heart failure; HHD, hypertensive heart disease; CHD-HF, heart failure and coronary heart disease; VHD, valvular heart disease (excluding rheumatic fever); CM, cardiomyopathy; IE, infective endocarditis; PHD, pulmonary heart disease.

**Supplementary Table 4.** Causal estimates of genetic proxied gut microbe abundance on the risks of cardiomyopathy subtypes based on two-sample MR analyses

| **exposure** | **outcome** | **method** | **nSNP** | **beta** | **OR (95%CI)** | **p value** | **q value** |
| --- | --- | --- | --- | --- | --- | --- | --- |
| family *Peptostreptococcaceae* | NICM | Wald ratio | 1 | -0.091 | 0.913 (0.522-1.597) | 0.750 | 0.919 |
| family *Oxalobacteraceae* | NICM | Wald ratio | 1 | -0.042 | 0.959 (0.700-1.312) | 0.792 | 0.919 |
| genus *Peptococcus* | NICM | Wald ratio | 1 | 0.024 | 1.025 (0.742-1.416) | 0.883 | 0.919 |
| genus *Intestinibacter* | NICM | Wald ratio | 1 | -0.127 | 0.881 (0.540-1.438) | 0.613 | 0.919 |
| genus *Enterorhabdus* | NICM | Wald ratio | 1 | 0.048 | 1.049 (0.734-1.498) | 0.794 | 0.919 |
| genus *Erysipelatoclostridium* | NICM | Wald ratio | 1 | 0.102 | 1.108 (0.734-1.673) | 0.626 | 0.919 |
| genus *Tyzzerella3* | NICM | Wald ratio | 1 | -0.249 | 0.780 (0.580-1.047) | 0.098 | 0.623 |
| order *Gastranaerophilales* | NICM | Wald ratio | 1 | -0.018 | 0.982 (0.724-1.331) | 0.906 | 0.919 |
| genus *Bifidobacterium* | NICM | IVW | 2 | -0.287 | 0.750 (0.579-0.973) | 0.030 | 0.287 |
| genus *Oxalobacter* | NICM | Wald ratio | 1 | -0.145 | 0.865 (0.645-1.161) | 0.335 | 0.795 |
| genus *Allisonella* | NICM | Wald ratio | 1 | 0.025 | 1.026 (0.814-1.292) | 0.830 | 0.919 |
| genus *Ruminococcaceae UCG009* | NICM | Wald ratio | 1 | 0.235 | 1.264 (0.888-1.801) | 0.194 | 0.736 |
| genus *Faecalibacterium* | NICM | Wald ratio | 1 | -0.206 | 0.814 (0.478-1.385) | 0.447 | 0.919 |
| genus *Streptococcus* | NICM | Wald ratio | 1 | -0.206 | 0.814 (0.456-1.451) | 0.485 | 0.919 |
| genus *Ruminococcaceae UCG013* | NICM | Wald ratio | 1 | -0.401 | 0.670 (0.365-1.229) | 0.196 | 0.736 |
| genus *Eubacterium coprostanoligenes group* | NICM | Wald ratio | 1 | -0.030 | 0.971 (0.549-1.718) | 0.919 | 0.919 |
| genus *Ruminococcus1* | NICM | Wald ratio | 1 | -0.308 | 0.735 (0.424-1.273) | 0.271 | 0.736 |
| genus *Ruminococcus torques group* | NICM | Wald ratio | 1 | 0.349 | 1.417 (0.785-2.558) | 0.247 | 0.736 |
| family *Peptostreptococcaceae* | PCM | Wald ratio | 1 | 0.179 | 1.195 (0.533-2.681) | 0.665 | 0.797 |
| family *Oxalobacteraceae* | PCM | Wald ratio | 1 | 0.500 | 1.648 (1.046-2.597) | 0.031 | 0.209 |
| genus *Peptococcus* | PCM | Wald ratio | 1 | -0.204 | 0.816 (0.509-1.307) | 0.397 | 0.637 |
| genus *Intestinibacter* | PCM | Wald ratio | 1 | 0.238 | 1.269 (0.625-2.578) | 0.510 | 0.692 |
| genus *Enterorhabdus* | PCM | Wald ratio | 1 | -0.097 | 0.908 (0.542-1.520) | 0.713 | 0.797 |
| genus *Erysipelatoclostridium* | PCM | Wald ratio | 1 | 0.284 | 1.328 (0.732-2.409) | 0.350 | 0.637 |
| genus *Tyzzerella3* | PCM | Wald ratio | 1 | -0.464 | 0.629 (0.410-0.963) | 0.033 | 0.209 |
| order *Gastranaerophilales* | PCM | Wald ratio | 1 | 0.353 | 1.423 (0.918-2.207) | 0.115 | 0.351 |
| genus *Bifidobacterium* | PCM | IVW | 2 | -0.276 | 0.759 (0.522-1.103) | 0.148 | 0.351 |
| genus *Oxalobacter* | PCM | Wald ratio | 1 | -0.025 | 0.976 (0.637-1.494) | 0.910 | 0.910 |
| genus *Allisonella* | PCM | Wald ratio | 1 | -0.032 | 0.968 (0.693-1.354) | 0.851 | 0.898 |
| genus *Ruminococcaceae UCG009* | PCM | Wald ratio | 1 | 0.724 | 2.063 (1.224-3.476) | 0.007 | 0.124 |
| genus *Faecalibacterium* | PCM | Wald ratio | 1 | -0.291 | 0.748 (0.346-1.614) | 0.459 | 0.670 |
| genus *Streptococcus* | PCM | Wald ratio | 1 | -0.355 | 0.701 (0.305-1.610) | 0.402 | 0.637 |
| genus *Ruminococcaceae UCG013* | PCM | Wald ratio | 1 | -0.736 | 0.479 (0.199-1.152) | 0.100 | 0.351 |
| genus *Eubacterium coprostanoligenes group* | PCM | Wald ratio | 1 | -0.572 | 0.564 (0.244-1.306) | 0.181 | 0.383 |
| genus *Ruminococcus1* | PCM | Wald ratio | 1 | -0.162 | 0.850 (0.383-1.886) | 0.690 | 0.797 |
| genus *Ruminococcus torques group* | PCM | Wald ratio | 1 | -0.731 | 0.481 (0.204-1.136) | 0.095 | 0.351 |
| family *Peptostreptococcaceae* | HCM | Wald ratio | 1 | -0.403 | 0.669 (0.136-3.283) | 0.620 | 0.917 |
| family *Oxalobacteraceae* | HCM | Wald ratio | 1 | 0.242 | 1.273 (0.522-3.104) | 0.595 | 0.917 |
| genus *Peptococcus* | HCM | Wald ratio | 1 | -0.326 | 0.722 (0.286-1.825) | 0.491 | 0.917 |
| genus *Intestinibacter* | HCM | Wald ratio | 1 | 1.821 | 6.178 (1.532-24.904) | 0.010 | 0.199 |
| genus *Enterorhabdus* | HCM | Wald ratio | 1 | -0.865 | 0.421 (0.153-1.160) | 0.094 | 0.598 |
| genus *Erysipelatoclostridium* | HCM | Wald ratio | 1 | -0.141 | 0.868 (0.269-2.799) | 0.813 | 0.917 |
| genus *Tyzzerella3* | HCM | Wald ratio | 1 | -0.312 | 0.732 (0.316-1.696) | 0.467 | 0.917 |
| order *Gastranaerophilales* | HCM | Wald ratio | 1 | 0.454 | 1.575 (0.665-3.726) | 0.302 | 0.917 |
| genus *Bifidobacterium* | HCM | IVW | 2 | -0.047 | 0.955 (0.458-1.991) | 0.901 | 0.917 |
| genus *Oxalobacter* | HCM | Wald ratio | 1 | -0.191 | 0.826 (0.357-1.913) | 0.656 | 0.917 |
| genus *Allisonella* | HCM | Wald ratio | 1 | 0.035 | 1.036 (0.535-2.006) | 0.917 | 0.917 |
| genus *Ruminococcaceae UCG009* | HCM | Wald ratio | 1 | 0.079 | 1.083 (0.395-2.966) | 0.877 | 0.917 |
| genus *Faecalibacterium* | HCM | Wald ratio | 1 | -0.178 | 0.837 (0.183-3.831) | 0.819 | 0.917 |
| genus *Streptococcus* | HCM | Wald ratio | 1 | -1.838 | 0.159 (0.031-0.828) | 0.029 | 0.274 |
| genus *Ruminococcaceae UCG013* | HCM | Wald ratio | 1 | 0.397 | 1.487 (0.264-8.382) | 0.653 | 0.917 |
| genus *Eubacterium coprostanoligenes group* | HCM | Wald ratio | 1 | 0.239 | 1.270 (0.242-6.657) | 0.778 | 0.917 |
| genus *Ruminococcus1* | HCM | Wald ratio | 1 | 0.988 | 2.687 (0.560-12.880) | 0.217 | 0.917 |
| genus *Ruminococcus torques group* | HCM | Wald ratio | 1 | -0.402 | 0.669 (0.124-3.613) | 0.641 | 0.917 |
| family *Peptostreptococcaceae* | HCM-HF | Wald ratio | 1 | -0.456 | 0.634 (0.052-7.736) | 0.721 | 0.905 |
| family *Oxalobacteraceae* | HCM-HF | Wald ratio | 1 | 1.406 | 4.079 (1.000-16.631) | 0.050 | 0.475 |
| genus *Peptococcus* | HCM-HF | Wald ratio | 1 | -0.380 | 0.684 (0.158-2.959) | 0.611 | 0.893 |
| genus *Intestinibacter* | HCM-HF | Wald ratio | 1 | 1.107 | 3.024 (0.338-27.042) | 0.322 | 0.733 |
| genus *Enterorhabdus* | HCM-HF | Wald ratio | 1 | -1.757 | 0.173 (0.035-0.851) | 0.031 | 0.475 |
| genus *Erysipelatoclostridium* | HCM-HF | Wald ratio | 1 | 0.839 | 2.314 (0.368-14.555) | 0.371 | 0.733 |
| genus *Tyzzerella3* | HCM-HF | Wald ratio | 1 | -0.591 | 0.554 (0.148-2.077) | 0.381 | 0.733 |
| order *Gastranaerophilales* | HCM-HF | Wald ratio | 1 | 0.617 | 1.853 (0.480-7.162) | 0.371 | 0.733 |
| genus *Bifidobacterium* | HCM-HF | IVW | 2 | -0.131 | 0.877 (0.276-2.790) | 0.824 | 0.905 |
| genus *Oxalobacter* | HCM-HF | Wald ratio | 1 | 0.080 | 1.084 (0.289-4.066) | 0.905 | 0.905 |
| genus *Allisonella* | HCM-HF | Wald ratio | 1 | 0.231 | 1.260 (0.446-3.559) | 0.662 | 0.899 |
| genus *Ruminococcaceae UCG009* | HCM-HF | Wald ratio | 1 | -0.241 | 0.786 (0.161-3.845) | 0.766 | 0.905 |
| genus *Faecalibacterium* | HCM-HF | Wald ratio | 1 | -0.870 | 0.419 (0.039-4.534) | 0.474 | 0.750 |
| genus *Streptococcus* | HCM-HF | Wald ratio | 1 | -2.160 | 0.115 (0.009-1.533) | 0.102 | 0.644 |
| genus *Ruminococcaceae UCG013* | HCM-HF | Wald ratio | 1 | 1.914 | 6.782 (0.446-103.058) | 0.168 | 0.733 |
| genus *Eubacterium coprostanoligenes group* | HCM-HF | Wald ratio | 1 | 0.953 | 2.595 (0.193-34.941) | 0.472 | 0.750 |
| genus *Ruminococcus1* | HCM-HF | Wald ratio | 1 | 1.307 | 3.694 (0.313-43.579) | 0.299 | 0.733 |
| genus *Ruminococcus torques group* | HCM-HF | Wald ratio | 1 | -1.172 | 0.310 (0.022-4.378) | 0.386 | 0.733 |
| family *Peptostreptococcaceae* | ACM | Wald ratio | 1 | 3.755 | 42.721 (0.460-3965.456) | 0.104 | 0.885 |
| family *Oxalobacteraceae* | ACM | Wald ratio | 1 | 1.475 | 4.372 (0.348-54.904) | 0.253 | 0.892 |
| genus *Peptococcus* | ACM | Wald ratio | 1 | -0.803 | 0.448 (0.031-6.524) | 0.557 | 0.910 |
| genus *Intestinibacter* | ACM | Wald ratio | 1 | -2.992 | 0.050 (0.001-2.661) | 0.140 | 0.885 |
| genus *Enterorhabdus* | ACM | Wald ratio | 1 | -1.328 | 0.265 (0.015-4.779) | 0.368 | 0.892 |
| genus *Erysipelatoclostridium* | ACM | Wald ratio | 1 | -0.901 | 0.406 (0.015-11.342) | 0.596 | 0.910 |
| genus *Tyzzerella3* | ACM | Wald ratio | 1 | -0.400 | 0.670 (0.061-7.382) | 0.744 | 0.910 |
| order *Gastranaerophilales* | ACM | Wald ratio | 1 | -0.873 | 0.418 (0.037-4.688) | 0.479 | 0.910 |
| genus *Bifidobacterium* | ACM | IVW | 2 | 0.222 | 1.248 (0.154-10.103) | 0.835 | 0.910 |
| genus *Oxalobacter* | ACM | Wald ratio | 1 | -0.432 | 0.649 (0.060-7.070) | 0.723 | 0.910 |
| genus *Allisonella* | ACM | Wald ratio | 1 | 0.454 | 1.574 (0.242-10.245) | 0.635 | 0.910 |
| genus *Ruminococcaceae UCG009* | ACM | Wald ratio | 1 | -0.121 | 0.886 (0.051-15.301) | 0.934 | 0.934 |
| genus *Faecalibacterium* | ACM | Wald ratio | 1 | -0.389 | 0.678 (0.008-54.623) | 0.862 | 0.910 |
| genus *Streptococcus* | ACM | Wald ratio | 1 | -1.975 | 0.139 (0.001-15.447) | 0.411 | 0.892 |
| genus *Ruminococcaceae UCG013* | ACM | Wald ratio | 1 | -2.389 | 0.092 (0.001-12.707) | 0.342 | 0.892 |
| genus *Eubacterium coprostanoligenes group* | ACM | Wald ratio | 1 | 4.644 | 103.961 (0.949-11386.008) | 0.053 | 0.885 |
| genus *Ruminococcus1* | ACM | Wald ratio | 1 | 1.826 | 6.211 (0.071-539.776) | 0.423 | 0.892 |
| genus *Ruminococcus torques group* | ACM | Wald ratio | 1 | 3.075 | 21.647 (0.176-2659.177) | 0.210 | 0.892 |

MR, Mendelian randomization; nSNP, number of single-nucleotide polymorphisms. IVW, inverse variance weighted (fixed effects); OR, odds ratio; CI, confidence interval; NICM, non-ischemic cardiomyopathy; PCM, primary cardiomyopathy; HCM, hypertrophic cardiomyopathy; HCM-HF, heart failure and hypertrophic cardiomyopathy; ACM, alcoholic cardiomyopathy.

**Supplementary Table 5.** Causal estimates of genetic proxied gut microbe abundance on the risks of valvular heart disease subtypes based on two-sample MR analyses

| **exposure** | **outcome** | **method** | **nSNP** | **beta** | **OR (95%CI)** | **p value** | **q value** |
| --- | --- | --- | --- | --- | --- | --- | --- |
| family *Peptostreptococcaceae* | NRVD | Wald ratio | 1 | 0.011 | 1.011 (0.682-1.499) | 0.958 | 0.958 |
| family *Oxalobacteraceae* | NRVD | Wald ratio | 1 | -0.148 | 0.862 (0.691-1.076) | 0.190 | 0.663 |
| genus *Peptococcus* | NRVD | Wald ratio | 1 | -0.139 | 0.870 (0.692-1.095) | 0.235 | 0.663 |
| genus *Intestinibacter* | NRVD | Wald ratio | 1 | -0.151 | 0.860 (0.609-1.214) | 0.391 | 0.663 |
| genus *Enterorhabdus* | NRVD | Wald ratio | 1 | -0.009 | 0.991 (0.772-1.274) | 0.947 | 0.958 |
| genus *Erysipelatoclostridium* | NRVD | Wald ratio | 1 | 0.114 | 1.121 (0.839-1.498) | 0.439 | 0.663 |
| genus *Tyzzerella3* | NRVD | Wald ratio | 1 | -0.157 | 0.855 (0.694-1.052) | 0.139 | 0.663 |
| order *Gastranaerophilales* | NRVD | Wald ratio | 1 | 0.103 | 1.108 (0.895-1.371) | 0.345 | 0.663 |
| genus *Bifidobacterium* | NRVD | IVW | 2 | -0.077 | 0.926 (0.771-1.111) | 0.408 | 0.663 |
| genus *Oxalobacter* | NRVD | Wald ratio | 1 | -0.120 | 0.887 (0.721-1.092) | 0.259 | 0.663 |
| genus *Allisonella* | NRVD | Wald ratio | 1 | -0.217 | 0.805 (0.683-0.948) | 0.009 | 0.177 |
| genus *RuminococcaceaeUCG009* | NRVD | Wald ratio | 1 | 0.112 | 1.118 (0.871-1.437) | 0.381 | 0.663 |
| genus *Faecalibacterium* | NRVD | Wald ratio | 1 | -0.138 | 0.871 (0.598-1.269) | 0.472 | 0.663 |
| genus *Streptococcus* | NRVD | Wald ratio | 1 | 0.110 | 1.117 (0.741-1.684) | 0.598 | 0.710 |
| genus *RuminococcaceaeUCG013* | NRVD | Wald ratio | 1 | 0.262 | 1.299 (0.847-1.992) | 0.230 | 0.663 |
| genus *Eubacteriumcoprostanoligenesgroup* | NRVD | Wald ratio | 1 | -0.141 | 0.869 (0.577-1.307) | 0.500 | 0.663 |
| genus *Ruminococcus1* | NRVD | Wald ratio | 1 | 0.203 | 1.226 (0.832-1.806) | 0.304 | 0.663 |
| genus *Ruminococcustorquesgroup* | NRVD | Wald ratio | 1 | -0.012 | 0.989 (0.651-1.500) | 0.957 | 0.958 |
| family *Peptostreptococcaceae* | RVD | Wald ratio | 1 | 0.022 | 1.022 (0.155-6.761) | 0.982 | 0.982 |
| family *Oxalobacteraceae* | RVD | Wald ratio | 1 | 0.220 | 1.246 (0.432-3.600) | 0.684 | 0.982 |
| genus *Peptococcus* | RVD | Wald ratio | 1 | 0.179 | 1.195 (0.398-3.590) | 0.750 | 0.982 |
| genus *Intestinibacter* | RVD | Wald ratio | 1 | -0.132 | 0.876 (0.168-4.565) | 0.875 | 0.982 |
| genus *Enterorhabdus* | RVD | Wald ratio | 1 | 0.140 | 1.150 (0.345-3.834) | 0.820 | 0.982 |
| genus *Erysipelatoclostridium* | RVD | Wald ratio | 1 | -0.036 | 0.965 (0.240-3.882) | 0.960 | 0.982 |
| genus *Tyzzerella3* | RVD | Wald ratio | 1 | -1.318 | 0.268 (0.098-0.730) | 0.010 | 0.191 |
| order *Gastranaerophilales* | RVD | Wald ratio | 1 | -0.274 | 0.761 (0.272-2.128) | 0.602 | 0.982 |
| genus *Bifidobacterium* | RVD | IVW | 2 | -0.308 | 0.735 (0.306-1.766) | 0.491 | 0.982 |
| genus *Oxalobacter* | RVD | Wald ratio | 1 | 0.372 | 1.450 (0.535-3.927) | 0.465 | 0.982 |
| genus *Allisonella* | RVD | Wald ratio | 1 | -0.184 | 0.832 (0.381-1.816) | 0.645 | 0.982 |
| genus *RuminococcaceaeUCG009* | RVD | Wald ratio | 1 | -0.181 | 0.834 (0.252-2.763) | 0.767 | 0.982 |
| genus *Faecalibacterium* | RVD | Wald ratio | 1 | -0.130 | 0.878 (0.145-5.307) | 0.887 | 0.982 |
| genus *Streptococcus* | RVD | Wald ratio | 1 | -1.758 | 0.172 (0.025-1.203) | 0.076 | 0.651 |
| genus *RuminococcaceaeUCG013* | RVD | Wald ratio | 1 | 1.701 | 5.481 (0.710-42.320) | 0.103 | 0.651 |
| genus *Eubacteriumcoprostanoligenesgroup* | RVD | Wald ratio | 1 | 1.403 | 4.067 (0.567-29.166) | 0.163 | 0.774 |
| genus *Ruminococcus1* | RVD | Wald ratio | 1 | -0.556 | 0.574 (0.089-3.682) | 0.558 | 0.982 |
| genus *Ruminococcustorquesgroup* | RVD | Wald ratio | 1 | -0.747 | 0.474 (0.064-3.491) | 0.463 | 0.982 |
| genus *Enterorhabdus* | CAVS | Wald ratio | 1 | -0.305 | 0.737 (0.515-1.056) | 0.097 | 0.426 |
| family *Peptostreptococcaceae* | CAVS | Wald ratio | 1 | -0.373 | 0.689 (0.391-1.212) | 0.196 | 0.619 |
| genus *Streptococcus* | CAVS | Wald ratio | 1 | 0.148 | 1.160 (0.643-2.092) | 0.623 | 0.796 |
| genus *Ruminococcustorquesgroup* | CAVS | Wald ratio | 1 | 0.483 | 1.621 (0.893-2.941) | 0.112 | 0.426 |
| order *Gastranaerophilales* | CAVS | Wald ratio | 1 | 0.007 | 1.007 (0.741-1.368) | 0.963 | 0.963 |
| genus *Peptococcus* | CAVS | Wald ratio | 1 | -0.406 | 0.666 (0.480-0.924) | 0.015 | 0.144 |
| genus *Bifidobacterium* | CAVS | IVW | 2 | 0.049 | 1.050 (0.809-1.364) | 0.712 | 0.796 |
| genus *Intestinibacter* | CAVS | Wald ratio | 1 | -0.112 | 0.894 (0.546-1.465) | 0.657 | 0.796 |
| family *Oxalobacteraceae* | CAVS | Wald ratio | 1 | -0.411 | 0.663 (0.483-0.910) | 0.011 | 0.144 |
| genus *RuminococcaceaeUCG009* | CAVS | Wald ratio | 1 | -0.044 | 0.957 (0.669-1.370) | 0.811 | 0.856 |
| genus *Faecalibacterium* | CAVS | Wald ratio | 1 | -0.128 | 0.880 (0.513-1.511) | 0.643 | 0.796 |
| genus *Ruminococcus1* | CAVS | Wald ratio | 1 | 0.271 | 1.312 (0.753-2.285) | 0.338 | 0.796 |
| genus *Allisonella* | CAVS | Wald ratio | 1 | -0.247 | 0.781 (0.618-0.987) | 0.038 | 0.242 |
| genus *Tyzzerella3* | CAVS | Wald ratio | 1 | -0.097 | 0.908 (0.674-1.222) | 0.524 | 0.796 |
| genus *RuminococcaceaeUCG013* | CAVS | Wald ratio | 1 | 0.226 | 1.253 (0.679-2.312) | 0.470 | 0.796 |
| genus *Oxalobacter* | CAVS | Wald ratio | 1 | -0.111 | 0.895 (0.665-1.206) | 0.467 | 0.796 |
| genus *Eubacteriumcoprostanoligenesgroup* | CAVS | Wald ratio | 1 | -0.257 | 0.773 (0.430-1.390) | 0.390 | 0.796 |
| genus *Erysipelatoclostridium* | CAVS | Wald ratio | 1 | 0.091 | 1.095 (0.724-1.658) | 0.667 | 0.796 |

MR, Mendelian randomization; nSNP, number of single-nucleotide polymorphisms. IVW, inverse variance weighted (fixed effects); OR, odds ratio; CI, confidence interval; NRVD, non-rheumatic valve diseases; RVD, rheumatic valve diseases; CAVS, calcific aortic valvular stenosis.
